# Prostatic artery embolization using reflux-control microcatheter: prospective experience addressing feasibility

**DOI:** 10.1186/s42155-022-00337-8

**Published:** 2022-12-07

**Authors:** André Moreira de Assis, Willian Yoshinori Kawakami, Airton Mota Moreira, Francisco Cesar Carnevale

**Affiliations:** Interventional Radiology Department, Radiology Institute, University of Sao Paulo Medical School, Dr. Enéas de Carvalho Aguiar Avenue, 255, Cerqueira César, São Paulo, 05403-000 Brazil

**Keywords:** Benign prostatic hyperplasia, Prostate artery embolization, Lower urinary tract symptoms, Non-target embolization

## Abstract

**Purpose:**

To evaluate the efficacy and safety of Prostatic Artery Embolization (PAE) using a reflux control microcatheter.

**Materials and methods:**

This is a prospective, single-center investigation that included 10 patients undergoing PAE for treatment of lower urinary tract symptoms (LUTS) attributed to benign prostate hyperplasia (BPH). Baseline, 3-month, and 12-month efficacy endpoints were obtained for all patients and included prostate-specific antigen (PSA), uroflowmetry, pelvic magnetic resonance imaging (MRI), and clinical assessment using the International Prostate Symptom Score (IPSS) questionnaire and the IPSS-Quality of life (QoL) item. Complications were assessed using the Cirse classification system.

**Results:**

Ten patients entered statistical analysis and presented with significant LUTS improvement 12 months after PAE, as follows: mean IPSS reduction of 86.6% (2.8 vs. 20.7, − 17.9, *P* < 0.001), mean QoL reduction of 79.4% (1.1 vs. 5.4, − 4.3, *P* < 0.001), mean prostatic volume reduction of 38.4% (69.3 cm^3^ vs. 112.5 cm^3^, − 43.2 cm^3^, *P* < 0.001), mean peak urinary flow (Qmax) increase of 199.4% (19.9 mL/s vs. 6.6 mL/s, + 13.3 mL/s, *P* = 0.006) and mean PSA reduction of 50.1% (3.0 ng/mL vs. 6.1 ng/mL, − 3.0 ng/mL, *P* < 0.001). One patient (10%) needed transurethral resection of the prostate (TURP) after PAE due to a ball-valve effect. One microcatheter (10%) needed to be replaced during PAE due to occlusion. Non-target embolization was not observed in the cohort.

**Conclusion:**

This initial experience suggests that PAE using a reflux control microcatheter is effective and safe for the treatment of LUTS attributed to BPH.

## Introduction

Prostatic artery embolization (PAE) is considered a minimally invasive procedure to treat lower urinary tract symptoms attributed to benign prostatic hyperplasia (LUTS/BPH), leading to improvement of all objective urological outcomes, as well as the quality of life of patients suffering from the disease (McWilliams et al, 2019; Wang et al, 2016; Uflacker et al, 2016; Malling et al, 2019; Feng et al, 2017; Pyo & Cho, 2017; Shim et al, 2017). Also, PAE presents with a favorable safety profile and is performed on an outpatient basis (Moreira et al, 2017).

Complications after PAE are usually infectious or due to non-target embolization (NTE) and are considered minor most of the time (Moreira et al, 2017). However, NTE such as bladder wall necrosis, hematochezia due to rectum ischemic ulcers and penile ulcers have been previously described (Pisco et al, 2016; Abt et al, 2018), even when specific measures such as coiling high-flow anastomosis are employed. During the procedure, avoiding reflux of the embolic agent to non-target structures, notably to the bladder and penis, is also a paramount technical concern (Moreira et al, 2017).

Other protective measures such as the use of reflux-controlling and balloon occlusion microcatheters were scarcely described so far and can potentially reduce the incidence of complications after PAE, specially NTE (Bilhim et al, 2019; Ayyagari et al, 2019). The aim of this study is to evaluate the feasibility, efficacy, and safety of PAE using a reflux control microcatheter in patients with LUTS / BPH.

## Materials and methods

This is a prospective, single-arm, single-center investigation that included 10 consecutive patients who underwent PAE to treat LUTS / BPH using the 2.4F SeQure® microcatheter (Guerbet, Paris, France). All participants signed written informed consent to participate in the investigation, and this study was approved by the institutional Ethics Committee (protocol number: 10373019.5.0000.0068). This manuscript was written based on the STROBE checklist for cohort studies (von Elm et al, 2008).

### Inclusion criteria

- LUTS / BPH refractory to medical treatment for at least 30 days (alpha-1-adrenergic receptor antagonist and/or 5-alpha-reductase inhibitor)
- International Prostate Symptom Score (IPSS) > 7 (moderate to severe symptoms).

### Exclusion criteria

- Histologic diagnosis of prostate cancer, neurogenic bladder disorders, or with a creatinine level > 2.0 mg/dL
- Large bladder diverticulum or stones with surgical indication
- Active urinary infection
- Prostate volume < 40 cm^3^

Baseline assessment included prostate MRI, pelvic US, uroflowmetry, clinical evaluation with IPSS and its subitem QoL, and serum PSA assessment. All patients returned for follow-up at 3 months and 12 months after PAE.

#### Pelvic MRI protocol

Pelvic MRI was performed either on a 1.5 or 3.0 Tesla magnet, using a phased pelvic array coil. A gadolinium-based contrast medium with a power injector at a dose of 0.1 mL/kg (or 0.2 mL/kg) was used, followed by 20 mL of saline flush. Prostatic measurements were obtained, and volume was calculated using the ellipsoid formula. The same specialist performed all imaging analyses before and after PAE. All periprostatic structures were carefully analyzed looking for NTE or other complications in follow-up MRI.

#### PAE procedure

All patients underwent PAE according to previously described methods (Carnevale et al, 2020a; Dias Jr et al, 2021). All procedures took place in a dedicated interventional radiology suite (Innova 3D CT; GE Healthcare, USA), using a nonionic contrast medium (320 mg/mL iodixanol; Visipaque; GE Healthcare, USA).

Procedures were performed under local anesthesia, through a unilateral right femoral approach. Selective 3D-angiography from the right and left internal iliac arteries was performed using a 5-F CPC catheter (Merit Medical, South Jordan, USA), to assess the blood supply to the prostate. Selective catheterization of the right and left prostatic arteries (PA) was performed using the 2.4-F SeQure® microcatheter (Guerbet, France). Anatomical findings were confirmed by Cone Beam CT (CBCT) using the EmboASSIST software (GE Healthcare, USA).

After selective injection of vasodilator in each PA, (isosorbide mononitrate 5 mg, Eurofarma, Brazil) embolization was performed with a combination of 100-300 (until near stasis) and 300-500 (until total stasis) micrometers Embosphere microspheres (Biosphere, Roissy, France), using the PErFecTED technique (Dias Jr et al, 2021). Each vial was diluted to 22 mL with equal parts of saline (10 mL) and contrast media (10 mL). All ten patients were discharged from the hospital 2-6 hours after the procedure.

#### Endpoints and follow-up protocol

Objective efficacy endpoints included peak urinary flow rate (Qmax, assessed by uroflowmetry), prostatic volume (assessed by MRI) and PSA. Patients’ clinical symptoms were assessed using the IPSS questionnaire and the IPSS-QoL item, for which responses range from “6, Terrible” to “0, Delighted”. All efficacy endpoints were obtained before PAE and at 3- and 12-month follow-up.

The Cirse classification system for complications (Filippiadis et al, 2017) was used to grade adverse events (grades I-VI). Adverse events including NTE were actively surveyed during the whole follow-up period, especially during the first month, by both presential and telephonic programmed contact with the patients.

#### Statistical analysis

Statistical analyses were conducted using GraphPad Prism 3.0 (San Diego, CA, EUA). Categorical variables were expressed as percentages, and numeric variables were described as means accompanied by standard deviations. The comparisons over time were performed using the Kruskal-Wallis test. Statistical significance was defined as a bicaudal type I error (*P*-value) < 0.05 for all analyses.

## Results

From February to October 2020, 10 patients who met the inclusion criteria underwent PAE. Nine patients (90%) had LUTS refractory to medical treatment (8 patients in the use of alpha-1-receptor antagonist and one patient in use of 5-alpha-reductase inhibitor), and one patient (10%) had intolerance to medical treatment (hypotension). One patient died during follow-up 6 months after PAE from an unrelated cause (lung cancer). His 3-month follow-up data were not excluded from statistical analysis. Patients’ baseline characteristics are summarized in Table 1.

**Table 1:** Baseline characteristics

	N	Mean ± STD	Range
Age (Years)	10	63.4 ± 5.6	52 – 72
IPSS	10	20.7 ± 5.7	12 – 29
QoL	10	5.4 ± 0.5	5 – 6
Qmax (mL/s)	10	6.6 ± 6.2	2.7 – 13.0
PV (cm^3^)	10	112.5 ± 35.4	46.5 – 130.1
PSA (ng/mL)	10	6.1 ± 2.8	0.6 – 7.0
ALPHA-BLOCKERS	10	8 (80%)	–
5-ARI INHIBITORS	10	1 (10%)	–

*STD* Standard deviation, *IPSS* International Prostate Symptoms Score, *QoL* Quality of life, *Qmax* Peak urinary flow rate, *PV* Prostatic volume, *PSA* Prostatic specific antigen

A total of 25 prostatic arterial branches were identified and embolized (double prostatic vascularization in 5/20 hemipelvis, 25%). According to the anatomic classification described by Assis et al. (de Assis et al, 2015), types of vascularization observed in this cohort were: Type I – 7/25 (28.0%), type II – 3/25 (12.0%), type III – 6/25 (24.0%), type IV – 7/25 (28.0%), and type 5 – 2/25 (8.0%).

Bilateral embolization of the PAs was performed in all 10 patients (100%) and the PErFecTED technique was possible in 24/25 prostatic branches (96.0%).The technical details of the procedures are described in Table 2. In one patient (10.0%), occlusion of the microcatheter was observed during embolization using 300-500 μm Embospheres, and microcatheter exchange was necessary to resume PAE.

**Table 2:** PAE procedure variables

	MEAN	STD
BILATERAL EMBOLIZATION	10 (100.0%)	–
CONTRAST MEDIUM VOLUME (ML)	184.6	74.7
FLUOROSCOPIC TIME (MIN)	66.8	22.7
DAP (cGy / cm^2^)	218.6	98.8
Peak skin dose (mGy)	1752.4	872.2
Total embolic volume (mL)	19.3	5.5

Categorical variables were described as absolute number and percentage

*DAP* Dose area product, *STD* Standard deviation

Nine out of the 10 patients (90%) presented with LUTS improvement, not needing any other specific treatment during follow-up. At the last follow-up evaluation (12 months), patients had a mean reduction of 17.9 points in IPSS, of 4.3 points in QoL, 38.4% in prostatic volume and 50.1% in PSA levels (*P* < 0.001 for all), while mean Qmax increased by 13.3 mL/s (199.4%, *P* = 0.006). The efficacy outcomes of the entire cohort are described on Table 3.

**Table 3:** PAE efficacy outcomes after 3 and 12 months

	baseline	3 months	change (%)	12 months	change (%)	*P*-value
IPSS	20.7 ± 5.7	2.3 ± 2.2	−18.4 (− 88.7%)	2.8 ± 3.8	−17.9 (− 86.6%)	< 0.001
QoL	5.4 ± 0.5	1.3 ± 1.8	−4.1 (− 75.9%)	1.1 ± 1.1	−4.3 (− 79.4%)	< 0.001
PV (cm^3^)	112.5 ± 35.4	75.8 ± 29.9	− 36.7 (− 32.6%)	69.3 ± 37.1	− 43.2 (− 38.4%)	< 0.001
Qmax (ml/s)	6.6 ± 6.2	22.5 ± 19.8	+ 15.8 (+ 238.0%)	19.9 ± 7.5	+ 13.3 (+ 199.4%)	0.006
PSA (ng/ml)	6.1 ± 2.8	2.2 ± 1.7	− 3.9 (− 64.9%)	3.0 ± 2.4	− 3.0 (− 50.1%)	< 0.001

Parametric variables described as means ± standard deviations

*IPSS* International Prostate Symptoms Score, *QoL* Quality of life, *Qmax* Peak urinary flow rate, *PV* Prostatic volume

**Fig. 1:**
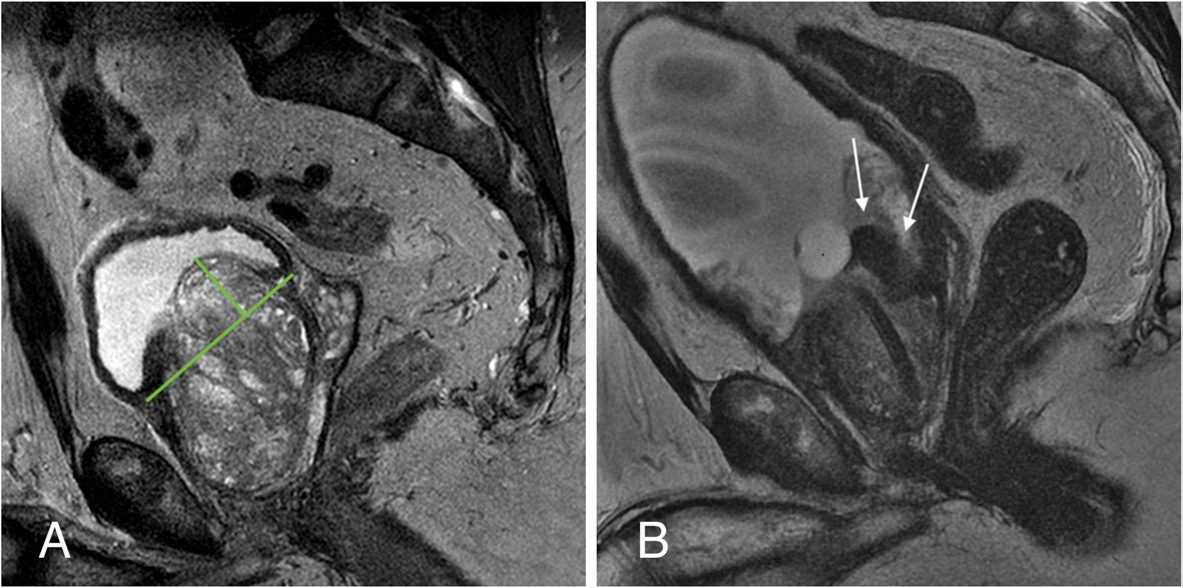
**A** Pre PAE T2-weighted MRI in sagittal view showing a very enlarged prostate, with a large medium lobe (grade III IPP = 26 mm). **B** After embolization, marked hypointense signal and volumetric reduction of the medium lobe was observed (arrows). (*) Foley balloon. A cystoscopy confirmed the presence of a necrotic medium lobe leading to ball-valve effect

All ten patients presented with mild dysuria, pelvic/perineal pain, and an increase in urinary frequency after PAE (100%), which were considered as expected collateral effects rather than complications (Moreira et al, 2017). One patient developed hematuria and early acute urinary retention a few days after PAE. MRI followed by cystoscopy was performed to further investigate, diagnosing a necrotic medium-lobe leading to ball-valve effect (Fig. 1), which was not considered to be related to the specific technique of embolization or the materials utilized. It required a transurethral resection of the necrotic medium lobe tissue, leading to the immediate resolution of the symptoms (grade III complication). No other complication including NTE was observed.

## Discussion

In the last decade, PAE gained ground as an alternative minimally invasive procedure for LUTS / BPH, much based on its favorable safety profile, and the possibility of being performed on an outpatient basis (McWilliams et al, 2019; Wang et al, 2016; Uflacker et al, 2016; Malling et al, 2019; Feng et al, 2017; Pyo & Cho, 2017; Shim et al, 2017). NTE, however, remains a primary source of concern, leading to the investigation of novel protective technologies.

The reflux-control mechanism of the SeQure® microcatheter involves the discharge of filtered contrast solution through a small side fenestrate, creating a fluid barrier that avoids the reflux of microspheres, which are delivered by the distal-end hole of the device; thus, the microcatheter utilizes fluid dynamics to minimize the risk of NTE (Rizzitelli et al, 2021). In that sense, reflux of contrast solution is expected and seen routinely during embolization using this microcatheter, while the risk of embolic particle reflux is mitigated.

**Fig. 2:**
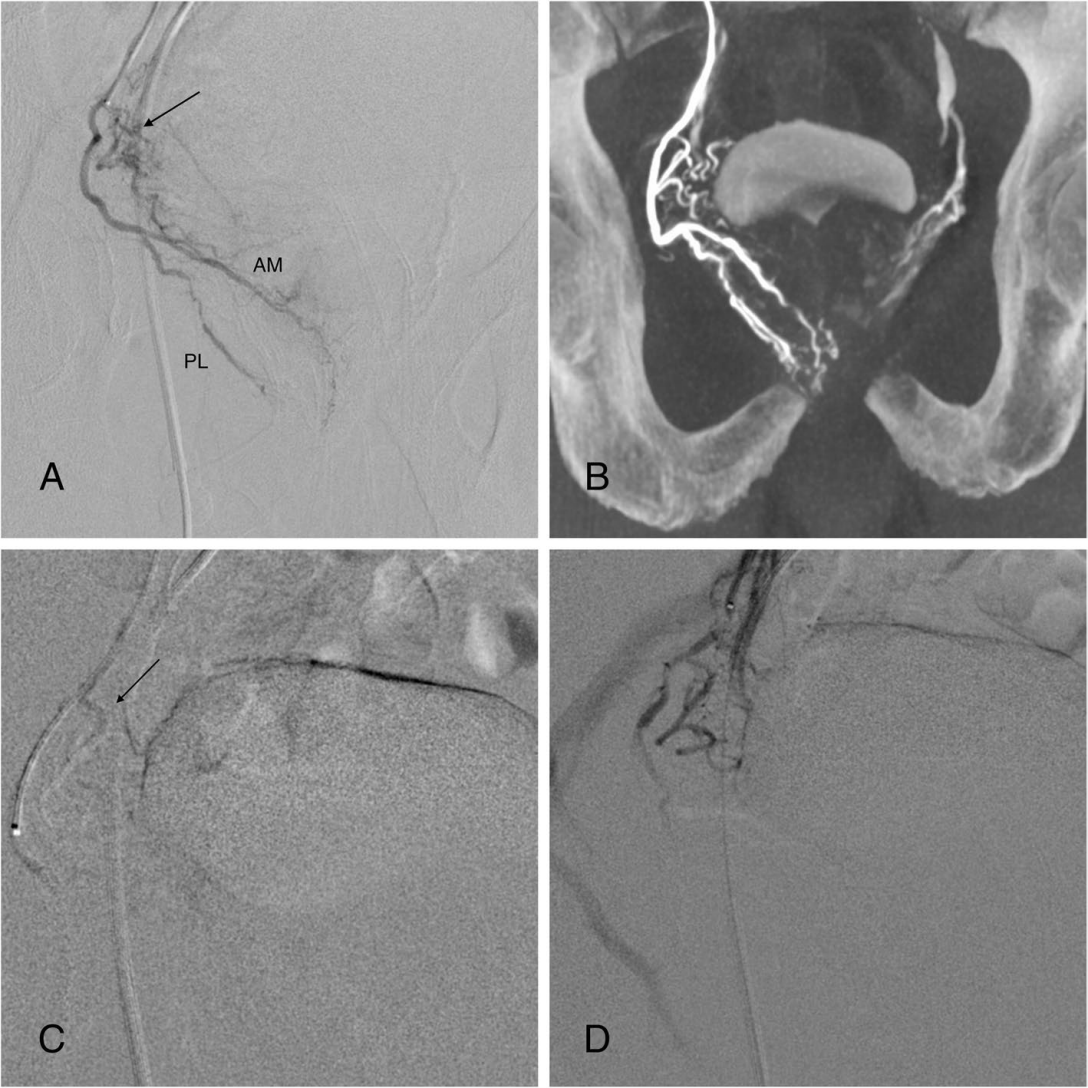
**A** Selective right prostatic artery DSA in oblique view showing the normal posterolateral (PL) and anteromedial (AM) prostatic branches. Cranially, a large branch to the bladder was also observed (arrow). **B** Intraprocedural Cone Beam CT in coronal view confirming the findings seen in DSA. **C** DSA while microspheres were injected demonstrating reflux to the bladder branch (arrow). **D** Final DSA after embolization showing devascularization of the prostatic branches and preservation of the bladder arterial branch

In 2021, a pre-clinical study (Rizzitelli et al, 2021) compared the outcomes of embolization using the SeQure® versus a standard end-hole microcatheter in 14 domestic pigs (28 kidneys). In this investigation, an automated injection protocol was used to ensure equivalent parameters. The embolization outcomes were assessed by angiography, gross pathology, and CT scans of the extracted kidneys. Although contrast reflux was visible in all cases during embolization, CT scans demonstrated more frequently areas of NTE of radiopaque microspheres in the standard microcatheter group in a qualitative analysis performed by 5 blinded interventional radiologists (least-square means of 3.8 vs. 3.2, *P* = 0.01). Similarly, in our study, all non-target branches were observed to be patent after embolization (Fig. 2).

In the present cohort, a strict clinical and radiological follow-up protocol including MRI showed that no clinical or subclinical NTE was observed, with a normal aspect of the periprostatic structures in all patients. Noteworthy that the microcatheter used potentially protects against microspheres reflux to non-target arterial branches; however, high-flow anastomosis to relevant structures should still be coiled to protect against NTE whenever present.

The grade III complication observed (ball-valve effect) occurred in a patient with a large prostate (130 cm^3^), and a 26 mm intraprostatic protrusion (grade III IPP) caused by a large, asymmetric medium lobe (Fig. 1). In fact, patients with a large medium lobe seem to be more frequently prone to complications after PAE. Meira et al. (Meira et al, 2021) recently reported the efficacy and safety of PAE in patients with different IPP grades, showing a higher overall incidence of complications in grade III IPP when compared with grade II (62.5% vs. 23.4% *P* ≤ 0.01). Also, all major complications observed in the cohort occurred in grade III IPP patients (3/32, 9.4%), which included ball-valve effect, hematuria needing cystoscopic management, and persistent urinary tract infection.

Using the 2.4-F reflux control microcatheter, bilateral embolization was possible in all 10 patients (100%), while deeper, intraprostatic navigation into the prostatic branches (PErFecTED technique) was possible in 24/25 prostatic branches (96.0%). Also, even in the type I PAs (7/25, 28%), which are considered as of harder catheterization (de Assis et al, 2015), no additional difficulty related to the material was observed. In one patient (10.0%), occlusion of the microcatheter was observed during the embolization of the first prostatic side with 300-500 μm Embospheres, and microcatheter exchange was necessary to resume PAE. After removing the microcatheter, occlusion of its distal end by impacted particles was observed, as well as exteriorization of embolic material through the damaged side holes during saline injection. Although we did not modify the dilution protocol after this episode, no other similar event was observed. It is possible that using a more diluted microsphere solution could help reduce the incidence of microcatheter occlusion and subsequent damage of the reflux control mechanism.

Regarding the efficacy outcomes, the improvements in IPSS and QoL scores and the objective endpoints such as Qmax, prostatic volume, and PSA levels were similar to those previously described in multiple single-center series and metanalyses (Wang et al, 2016; Uflacker et al, 2016; Malling et al, 2019; Feng et al, 2017; Pyo and Cho 2017; Shim et al, 2017; Carnevale  et al, 2020b; Bagla et al, 2014) and lasted during the 12-month follow-up (Table 3). In 2019, a clinical trial comparing the outcomes of PAE using conventional (cPAE) versus balloon-occlusion microcatheter (bPAE) showed no difference in the efficacy outcomes between groups (Bilhim et al, 2019). Although coiling was employed as a protective measure in both arms (8.7% in the bPAE group and 14.0% in the cPAE group, *P* = 0.51), NTE resulting in penile lesions (*n* = 3, 7.0%) and rectal bleeding (*n* = 2, 4.7%) occurred only in the cPAE group. Similarly, we did not observe any clinical or subclinical NTE event.

Limitations of the present investigation include its small sample size, leading to higher variability of the results and possible bias. Also, it does not allow a definitive conclusion about the extent of the protection against NTE. Even so, it was possible to obtain statistical significance for all the intended endpoints using non-parametric tests. Also, the findings obtained were exclusively compared to historical data, in which populations are not necessarily similar regarding baseline aspects such as prostatic volume, arterial anatomy, and technique utilized, among others. Larger prospective trials would be more suitable to address the efficacy of PAE using the reflux-control microcatheter. Finally, no long-term data was obtained, although the short- and medium-term results presented were considered enough to attest to the feasibility of the method, even using a 2.4-F microcatheter, which is often considered too large for PAE.

In conclusion, this initial experience suggests that PAE using the reflux control microcatheter is effective and safe for the treatment of LUTS / BPH. No NTE was observed during follow-up.

## Data Availability

Please contact author for data requests.

## Abbreviations

bPAE: Prostatic artery embolization using balloon-occlusion microcatheter
BPH: Benign prostatic hyperplasia
cPAE: Prostatic artery embolization using conventional microcatheter
CBCT: Cone beam computed tomography
DAP: Dose area product
DSA: Digital subtraction angiography
F: French
IPP: Intraprostatic protrusion
IPSS: International prostate symptom score
LUTS: Lower urinary tract symptoms
MRI: Magnetic resonance imaging
NTE: Non-target embolization
PA: Prostatic artery
PAE: Prostatic artery embolization
PErFecTED: Proximal embolization first, then embolize distal
PSA: Prostatic specific antigen
Qmax: Peak urinary flow
QoL: Quality of life score
STD: Standard deviation
STROBE: Strengthening the reporting of observational studies in epidemiology
TURP: Transurethral resection of the prostate
US: Ultrasound
